# Psychological stress and influence factors in elderly patients with mild coronary heart disease: a longitudinal follow-up study in Shanghai, China

**DOI:** 10.3389/fpsyg.2024.1399061

**Published:** 2024-12-11

**Authors:** Yunwei Zhang, Qiyong Wu, Qiaotao Xie, Zhimin Xu, Xiuhui Yang, Yashuang Luo, Lingshan Wan, Ya Yang, Yibo Wang, Hansheng Ding

**Affiliations:** ^1^Shanghai Health Development Research Center (Shanghai Medical Information Center), Shanghai, China; ^2^Department of Thoracic and Cardiac Surgery, The Affiliated Changzhou No. 2 People’s Hospital of Nanjing Medical University, Changzhou, Jiangsu, China; ^3^Luohe Central Hospital, Luohe, Henan, China; ^4^Xinhua Hospital Affiliated to Shanghai Jiaotong University School of Medicine, Shanghai, China; ^5^Dahua Hospital, Shanghai, China; ^6^Department of Cardiology, Huangpu Branch of Ninth People’s Hospital, Shanghai Jiao Tong University School of Medicine, Shanghai, China

**Keywords:** coronary heart disease, elderly care, health status, psychological stress, sleep condition

## Abstract

**Introduction:**

Effective health management is crucial for elderly patients with coronary heart disease (CHD). This study applied a Psycho-Cardiology model to CHD management, aiming to assess psychological stress among patients with mild CHD and identify potential influencing factors to provide substantiating evidence.

**Methods:**

This longitudinal study was based on a 9-year follow-up program of a community population in Shanghai, China. A total of 44,552 elderly people were included, with the average age being 74.9 (±10.35) years, and the proportion of female participants being 56.5%. To evaluate and compare the effect of the disease, individuals were categorized into four groups based on their medical records from the past 6 months, these being (I) a CHD with other chronic diseases group, (II) a CHD only group, (III) non-CHD patients with one (or more) chronic disease group, and (IV) non-patient group. Demographic characteristics, sleep quality and health status of each participants were collected using the Unified Needs Assessment Form for Elderly Care Questionnaire. A multivariate logistic regression was used for statistic analysis.

**Results:**

Demographic characteristics differed significantly between the three chronic disease groups (Groups I, II and III) and the non-patient group. Participants in the CHD group reported poorer sleep quality, worse health status, and a more rapid health decline when compared to those with other chronic diseases. Factors such as age, gender, education level, disease duration, and family support were identified as potential influences on the self-reported subjective sleep quality in patients with mild CHD. While age, education level, living status and family support were potential factors influencing the self-assessed health status in participants without CHD (Groups III and IV).

**Conclusion:**

Patients with mild CHD may experience lower subjective sleep quality, health status scores, and a faster health-sleep decline, indicating elevated psychological stress. Higher education levels offer a protective effect against this stress, highlighting the importance of psycho-emotional interventions and educational strategies. Additionally, it is important to prioritize early intervention for newly diagnosed cases to aid in illness acceptance. These findings provide crucial insights for managing patients with mild CHD and inform the efficient allocation of healthcare resources.

## Introduction

Coronary heart disease (CHD) is a leading cause of morbidity and mortality worldwide, particularly among older adults (Lozano et al., 2013). Defined as a condition in which the coronary arteries are narrowed or blocked due to atherosclerosis, CHD significantly impairs blood flow to the heart and increases the risk of major cardiovascular events. According to data from the Annual Report on Cardiovascular Health and Diseases in China (2022) (The Writing Committee of the Report on Cardiovascular Health and Diseases in China and Hu, 2023), over 11.39 million individuals aged 60 and above are affected by CHD in the country.

Psychological factors, including stress, depression, and anxiety, play a critical role in the prognosis of CHD (Xu et al., 2023). It encompasses emotional and physiological responses to perceived threats or challenges, which can manifest through elevated blood pressure, increased heart rate, and heightened systemic inflammation (Munakata, 2018). Studies have shown that psychological stress at work can significantly exacerbate cardiac symptoms and increase the risk of adverse cardiovascular outcomes (Vaccarino and Bremner, 2024; Niedhammer et al., 2021). For example, mental stress-induced ischemia, has been linked to a higher risk of cardiovascular mortality and non-fatal myocardial infarction (Mehta et al., 2022). Persistent psychological distress has also been associated with a two-to four-fold increase in the risk of cardiovascular death among CHD patients (Stewart et al., 2017). Moreover, comorbid depression in CHD patients is associated with worse health outcomes, including reduced quality of life, increased emergency hospitalizations, and higher healthcare costs (Carney and Freedland, 2017; Xu et al., 2024).

These physiological responses are further aggravated by emotional and behavioral symptoms, such as sleep disturbances and reduced self-efficacy, which can further exacerbate the disease’s impact. Furthermore, sleep disturbances are closely linked to an increased risk of cardiovascular disease (CVD). Meta-analyses have identified somnipathy as an independent risk factor for CVD (Yang et al., 2022), while cohort studies have explored the long-term impact of sleep disorders in CHD patients (Khazaie et al., 2023). Psychological stress often exacerbates sleep problems, creating a negative feedback loop that worsens both emotional and physical health (Cappuccio et al., 2010). External symptoms, such as poor sleep quality and reduced self-efficacy, can further compromise CHD management and negatively affect clinical outcomes (Huang et al., 2024; Wang et al., 2016).

Existing studies have largely focused on severe CHD cases or short-term impacts (Gao et al., 2022; Moazzami et al., 2023), leaving a limited understanding of how psychological stress and related factors influence the long-term outcomes of elderly patients with mild CHD. Gregory et al. noted that nearly all evidence linking psychological disorders to prognostic outcomes derives from observational studies, which do not provide a strong basis for causal inference (Davidson et al., 2018). This highlights the need for long-term, large-sample cohort studies to strengthen the evidence base and to fill in the gaps. In recent years, limited research has targeted stable CHD patients, particularly within the Psycho-Cardiology model (Ge et al., 2022). Some studies have examined the effect of psychological stress on cardiovascular events or all-cause mortality among stable CHD patients (Pimple et al., 2019; Stewart et al., 2017), offering tertiary prevention strategies. However, evidence and strategies informed by primary and secondary prevention concepts often yield broader impacts and greater health benefits. Therefore, it is important to explore the roles of demographic variables and lifestyle factors on psychological stress and CHD progression in mild and stable CHD patients. Addressing these research gaps is crucial for developing evidence-based management strategies and early prevention approaches.

The present study aims to examine the prevalence and progression of psychological stress in elderly patients with mild CHD over a long-term follow-up. It seeks to identify key demographic and lifestyle predictors of psychological stress and investigate how these factors, alongside comorbid conditions, influence cardiac outcomes. By providing a comprehensive analysis, this research will support the effective integration of psychological care into CHD management, enhance the efficient allocation of healthcare resources and ultimately improve patients’ quality of life.

## Methods

### Design

This study was designed based on a longitudinal follow-up community population conducted in Shanghai, China, from 2013 to 2021. Participants were selected using a multistage sampling method. First, Jing’an District was selected through a random process from among the 16 administrative districts in Shanghai. Second, Jiangning Street Community was randomly designated from the 13 communities located within the boundaries of Jing’an District; Finally, a cohort of approximately 20,000 individuals aged 60 years and older underwent annual questionnaire-based assessments. This cohort encompassed older persons residing in their homes within the community as well as those residing in institutional settings.

To ensure the integrity and quality of the data collected, all investigators underwent a comprehensive training program before the survey began. The research protocol, bearing the reference number 2022009, obtained official approval from the Ethics Committee of the Shanghai Health Development Research Center in 2010. Additionally, all participants provided their written informed consent before participation, signifying their voluntary commitment to the research.

### Study population

**Figure 1:**
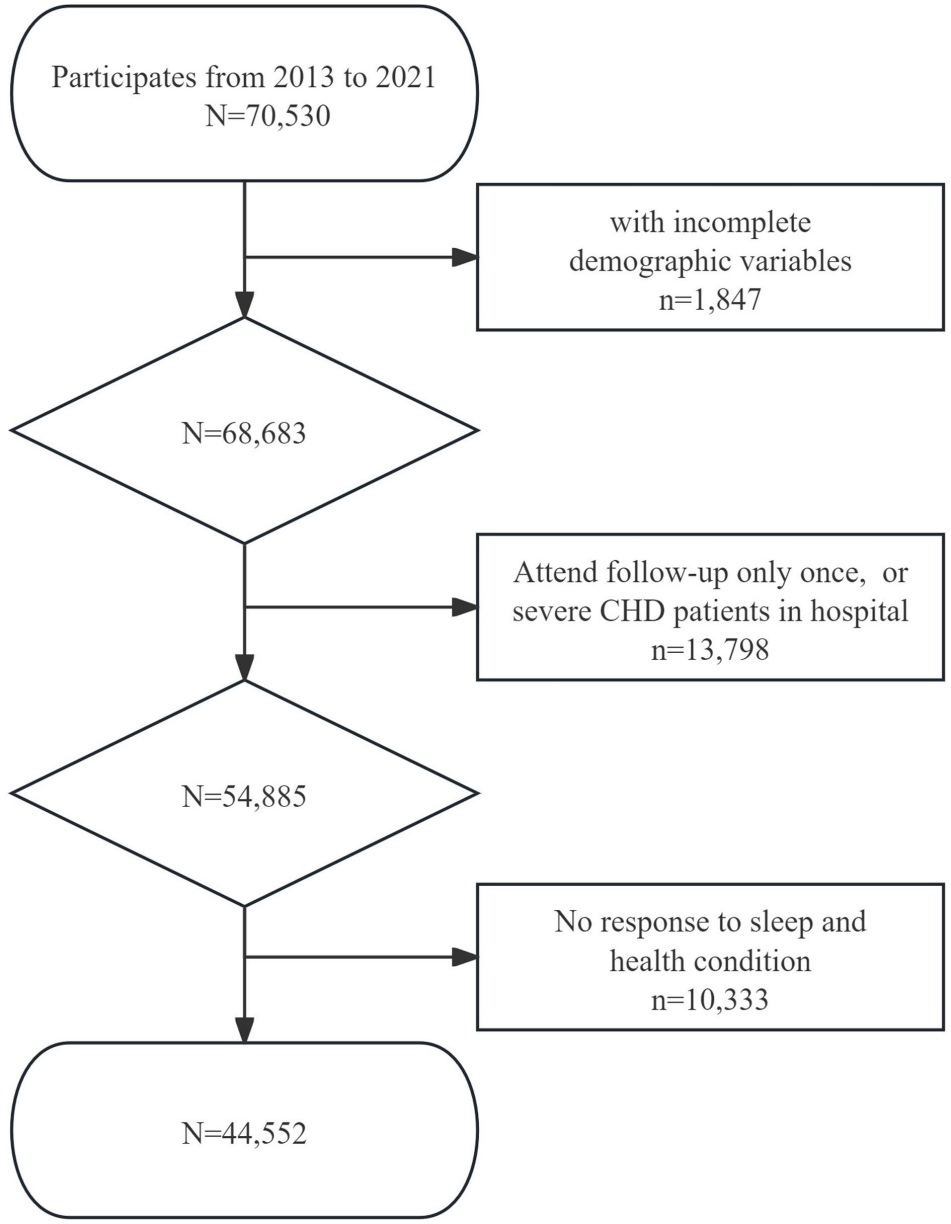
Flowchart of inclusion process of participates.

The inclusion criteria for this study were as follows: (1) aged 60 and above; (2) had continuous participation in the survey; (3) responses provided to the question, “How is your sleep condition at night?”; and (4) responses provided to the question “How do you consider your physical health to be?”. The exit criteria were: (1) elderly people withdraw from investigation such as relocation, death, or other reasons; (2) elderly patients with severe CHD who where hospitalized; and (3) participants who did not respond to questions about sleep or health status. Given the study’s open cohort design, participants who joined the cohort in different years were consolidated into a unified database. The primary focus was to examine the dynamics of key research variables, including self-reported subjective sleep quality and overall health condition. As a result, a total of 44,552 older adults were incorporated into the current study (Figure 1).

### CHD and other chronic disease groups

Data on symptoms, physical signs, laboratory tests, and physical examinations was collected by family doctors who were well-acquainted with the elderly person’s health status. The severity of CHD was classified according to the Chinese Elderly Coronary Heart Disease Chronic Disease Management Guidelines (2023), distinguishing between stable CHD and acute coronary syndrome (ACS). Criteria in this guidelines is similar with the Merck manuals. Finally, this study included only patients with stable CHD.

To compare the psychological impact of mild CHD and other non-fatally chronic ailments, this study included three typical chronic disease, chronic pneumonia, diabetes, and hypertension. The evaluation of all chronic diseases was based on medical records from the past 6 months and with evaluations performed by family physicians who possessed an intimate knowledge of the participants’ medical histories. The total of 44,552 participants was stratified into four groups as follows: (I) a CHD with other chronic diseases group, which included individuals with CHD and one or more of the other three chronic diseases. (II) a CHD only group, consisted of participants diagnosed exclusively with CHD. (III) non-CHD patients with one (or more) chronic disease group, included individuals with one or more the other three chronic diseases but were without CHD. (IV) non-patient group, which included participants who did not have any of the three chronic diseases at any of the follow-up stages.

### Assessment of sleep and health status

Self-reported subjective sleep quality and self-assessed health status were used as indirect measures of psychological stress. and were categorized into five point: point 5 denoted “very good sleep quality” or “very good health”, point 4 denoted “good sleep quality” or “good health”, point 3 denoted “general sleep quality” or “general health”, point 2 denoted “poor sleep quality” or “poor health”, and point 1 denoted “very poor sleep quality” or “very poor health”. This study classified sleep and health conditions using Likert scale, according to a widely used scale, the interRAI long-term care Screener (Version 9.1).

### Demographic characteristics

Demographic characteristics were collected through the Unified Needs Assessment Form for Elderly Care Questionnaire, which aimed to analyze the potential influence factors of psychological stress. Investigators gathered data on age, gender, education, living status, and family support through self-reports from participants or with assistance from their caregivers. “Living status” was determined based on responses to the question “Who do you live with?”, category 1 indicates “living alone”, category 2 indicates “living with spouse or other family members”, category 3 indicates “living with others”, typically in care institutions or at home with caregivers. “Family support” was determined based on responses to the question “What do you think of your family’s support”, category 1 represents “adequate material and emotional support”, category 2 represents “only material or emotional support”, category 3 represents “no material nor emotional support”, group 4 represents “no family or other condition”. This study classified demographic variables, using Likert scale, according to a widely used scale, the interRAI long-term care Screener (Version 9.1). In the two CHD groups (group I and group II), “disease duration” referred to the duration in years that participants had been affected by CHD as of the end of the follow-up period.

### Statistical analysis

Firstly, pairwise comparative analysis was used to investigate differences in demographic characteristics, self-reported subjective sleep quality and health status between each of three patient groups (group I, II and III) and the non-patient group (group IV). Secondly, multiple comparative analysis was employed using Dunnett *t* test, to examine the difference in demographic characteristics, self-reported subjective sleep quality and health condition across multiple groups. Variables that demonstrating statistical significance in the multiple comparative analysis were subsequently included in multivariate logistic regression model for the examination of influence factors. Collinearity diagnosis was conducted to ensure no significant collinearity among the independent variables. A significance level of *p* < 0.05 was considered as statistically significant. All data analyses were conducted using SAS 9.4 software (SAS Institute Inc., Cary, NC, United States).

## Results

### Demographic characteristics of participants

The analysis encompassed a total of 44,552 participants, and the mean and median follow-up period for data collection were 4.23 years and 4 years, respectively. Age, gender, education, living status, and family support were included. Almost all demographic characteristics were significantly different between chronic disease group (group I, II, and III) and non-patient group (group IV) (Table 1). Compared to the non-patient group, the chronic disease group exhibited several distinguishing features, including older age, lower education level, a lower proportion of cohabiting with spouse or other family members, and less family support. Furthermore, a significantly higher proportion of females was evident among the participants with CHD.

**Table 1:** Demographic characteristics of participants.

Characteristics	Group I ^*^	Group II ^*^	Group III ^*^	Group IV ^*^	Pairwise			Dunnett
	(*n* = 10,103)	(*n* = 2,290)	(*n* = 20,576)	(*n* = 11,583)	*p* ^a^	*p* ^b^	*p* ^c^	*p* ^d^
Age	81.36 ± 8.34	78.19 ± 10.27	74.36 ± 9.93	69.57 ± 9.35	<0.001	<0.001	<0.001	<0.001
Gender					<0.001	<0.001	0.086	<0.001
Male	3,752 (37.14)	880 (38.43)	9,356 (45.47)	5,382 (46.46)				
Female	6,351 (62.86)	1,410 (61.57)	11,220 (54.53)	6,201 (53.54)				
Education					<0.001	<0.001	<0.001	<0.001
≤6 years	6,958 (77.28)	1,386 (66.89)	11,185 (59.40)	4,553 (43.01)				
7–12 years	1,375 (15.27)	488 (23.55)	5,691 (30.22)	4,570 (43.17)				
>12 years	671 (7.45)	198 (9.56)	1,953 (10.37)	1,464 (13.83)				
Living status					<0.001	<0.001	<0.001	<0.001
Alone	1,218 (12.21)	284 (12.56)	2,025 (9.95)	1,150 (10.01)				
With spouse or other family members	3,093 (31.00)	981 (43.39)	10,912 (53.64)	7,724 (67.24)				
With others	5,667 (56.79)	996 (44.05)	7,407 (36.41)	2,613 (22.75)				
Family support					<0.001	0.020	<0.001	<0.001
Adequate material and emotional support	5,727 (71.70)	1,248 (71.52)	9,712 (71.70)	5,575 (73.98)				
Only material or emotional support	1,783 (22.32)	419 (24.01)	3,281 (24.22)	1,720 (22.82)				
No material nor emotional support	260 (3.25)	41 (2.35)	306 (2.26)	112 (1.49)				
No family or other condition	218 (2.73)	37 (2.12)	247 (1.82)	129 (1.71)				

* Group I = a CHD with other chronic diseases group, Group II = a CHD only group, Group III = non-CHD patients with one (or more) chronic disease group, Group IV = non-patient group. CHD, coronary heart disease.

a *p value of difference between Group I and Group IV using a pairwise test.*

b *p value of difference between Group II and Group IV using a pairwise test.*

c *p value of difference between Group III and Group IV using a pairwise test.*

d *p value of multiple comparisons using Dunnett t test.*

### Self-reported of sleep and health status in different groups

Table 2 presents the result of self-reported scores related to sleep and health status across different groups. Individuals in a CHD with other chronic diseases group (group I) reported the lowest self-assessed scores in both sleep and health status. In contrast, the non-patient group (group IV) displayed the highest scores. Notably, participants in a CHD only group (group II) reported significantly worse sleep quality and health status compared to non-CHD patients with one (or more) chronic disease group (group III) (*p* < 0.05).

**Table 2:** Self-reported of sleep and health status in different groups.

Characteristics	Group I ^*^	Group II ^*^	Group III ^*^	Group IV ^*^	Pairwise			Dunnett
	(*n* = 10,103)	(*n* = 2,290)	(*n* = 20,576)	(*n* = 11,583)	*p* ^a^	*p* ^b^	*p* ^c^	*p* ^d^
Sleep condition	2.64 ± 1.01	2.79 ± 1.05	2.92 ± 1.05	3.10 ± 1.13	<0.001	<0.001	<0.001	<0.001
Health status	2.90 ± 0.84	3.28 ± 0.80	3.36 ± 0.89	3.76 ± 0.83	<0.001	<0.001	<0.001	<0.001

* Group I = a CHD with other chronic diseases group, Group II = a CHD only group, Group III = non-CHD patients with one (or more) chronic disease group, Group IV = non-patient group. CHD, coronary heart disease.

a *p value of difference between Group I and Group IV using a pairwise test.*

b *p value of difference between Group II and Group IV using a pairwise test.*

c *p value of difference between Group III and Group IV using a pairwise test.*

d *p value of multiple comparisons using Dunnett t test.*

### Changes of sleep and health status in different groups

Table 3 presents the result of changes in self-reported subjective sleep quality and health status. A notable higher proportion of participants reporting a decline score among participants with CHD (group I and II). It is noteworthy that, across all the groups, the rate of decline in self-reported subjective sleep quality exceeded that of health status. Additionally, participants in a CHD only group (group II) exhibited a more rapid rate of decline when compared to the participants without CHD (group III and IV).

**Table 3:** Change speed of sleep and health status in different groups.

Characteristics	Group I ^*^		Group II ^*^		Group III ^*^		Group IV ^*^	
	(*n* = 10,103)		(*n* = 2,290)		(*n* = 20,576)		(*n* = 11,583)	
	Proportion	Change speed	Proportion	Change speed	Proportion	Change speed	Proportion	Change speed
Sleep condition	49.4%	−0.43 ± 0.68	49.3%	−0.45 ± 0.73	46.3%	−0.37 ± 0.61	42.8%	−0.42 ± 0.73
Health status	44.0%	−0.26 ± 0.48	43.5%	−0.29 ± 0.53	42.6%	−0.27 ± 0.46	42.2%	−0.28 ± 0.47

* Group I = a CHD with other chronic diseases group, Group II = a CHD only group, Group III = non-CHD patients with one (or more) chronic disease group, Group IV = non-patient group. CHD, coronary heart disease.

### Influence factors of self-reported subjective sleep quality

Regression analysis was conducted to explore the influence factors self-reported subjective sleep quality. Model incorporated variables that had demonstrated statistical significance in previous multiple comparisons analysis, which included age, gender, education, disease duration, living status, and family support. Results indicated that, across all groups, age exhibited a negative relationship with self-reported subjective sleep quality, while disease duration displayed a positive relationship with self-reported subjective sleep quality in participants with CHD (group I and II). Furthermore, logistic regression was conducted to explore the influence of gender, education, living status, and family support on sleep condition, and using the “very poor” category as the reference group. Findings suggested that female participants among a CHD with other chronic diseases group (group I) may experience better sleep condition. Additionally, individuals with higher levels of education may enjoy better sleep condition in group I, III and IV, although this trend was not observed in a CHD only group (group II). Participants who living with spouse or other family members demonstrated superior sleep condition compared to those living alone in non-CHD patients with one (or more) chronic disease group (group III) (*p* < 0.05) (Table 4).

**Table 4:** Influence factors of self-reported subjective sleep quality using logistic regression.

Characteristics	Group I ^*^		Group II ^*^		Group III ^*^		Group IV ^*^	
	(*n* = 10,103)		(*n* = 2,290)		(*n* = 20,576)		(*n* = 11,583)	
	*OR*	95%*CI*	*OR*	95%*CI*	*OR*	95%*CI*	*OR*	95%*CI*
Age	0.98	0.97–0.99	0.96	0.94–0.98	0.98	0.97–0.98	0.97	0.97–0.98
Gender								
Male	1.00	—	1.00	—	1.00	—	1.00	—
Female	1.23	1.01–1.51	0.71	0.50–1.02	1.03	0.96–1.10	0.99	0.91–1.09
Education								
≤6 years	1.00	—	1.00	—	1.00	—	1.00	—
7–12 years	1.47	1.08–2.01	1.01	0.63–1.60	1.19	1.10–1.29	1.35	1.22–1.50
>12 years	1.68	1.17–2.42	1.05	0.60–1.85	1.40	1.25–1.56	1.33	1.16–1.53
Disease duration	1.24	1.15–1.33	1.21	1.06–1.38	—	—	—	—
Living status								
Alone	1.00	—	1.00	—	1.00	—	1.00	—
With spouse or other family members	1.40	0.93–2.12	1.36	0.68–2.71	1.22	1.09–1.38	1.16	0.99–1.36
With others	1.14	0.78–1.69	0.69	0.35–1.36	1.01	0.90–1.14	0.89	0.75–1.05
Family support								
Adequate material and emotional support	1.00	—	1.00	—	1.00	—	1.00	—
Only material or emotional support	1.39	1.08–1.79	0.77	0.51–1.16	0.94	0.87–1.02	0.98	0.88–1.08
No material nor emotional support	0.86	0.41–1.98	0.73	0.31–1.69	0.87	0.70–1.09	0.85	0.57–1.27
No family or other condition	1.91	0.93–3.95	0.23	0.05–1.07	1.04	0.80–1.35	1.55	1.08–2.23

* Group I = a CHD with other chronic diseases group, Group II = a CHD only group, Group III = non-CHD patients with one (or more) chronic disease group, Group IV = non-patient group. CHD, coronary heart disease. Highlights in this table indicated that the effect size has reached a statistically significant level (*p* < 0.05).

### Influence factors of self-assessed health status

The same set of variables and regression model was conducted to assess the factors affecting self-assessed health status as in the analysis of sleep condition. The results revealed a consistent negative relationship between age and self-assessed health status across all groups. However, disease duration did not exhibit a statistically significant association with health status. In group III and IV, individuals with higher levels of education may experience better health status, while those residing in care institutions (living with others) might even experience worse health status than individuals living alone, and participants in group III showed a more significant negative correlation than group IV (*OR*: 0.58 vs. 0.70, *p* < 0.05). Additionally, among the participants without CHD (group III and IV), who lacked adequate family support may report worse health status, and non-patient group (group IV) showed a more significant negative correlation than non-CHD patients with one (or more) chronic disease group (group III) (*OR*: 0.23 vs. 0.44, *p* < 0.05) (Table 5).

**Table 5:** Influence factors of self-assessed health status using logistic regression.

Characteristics	Group I ^*^		Group II ^*^		Group III ^*^		Group IV ^*^	
	(*n* = 10,103)		(*n* = 2,290)		(*n* = 20,576)		(*n* = 11,583)	
	*OR*	95%*CI*	*OR*	95%*CI*	*OR*	95%*CI*	*OR*	95%*CI*
Age	0.97	0.96–0.99	0.97	0.94–0.99	0.95	0.94–0.95	0.95	0.94–0.96
Gender								
Male	1.00	—	1.00	—	1.00	—	1.00	—
Female	1.14	0.92–1.41	1.19	0.80–1.76	0.90	0.84–0.96	0.85	0.77–0.93
Education								
≤6 years	1.00	—	1.00	—	1.00	—	1.00	—
7–12 years	0.99	0.91–1.39	1.01	0.61–1.66	1.42	1.31–1.54	1.69	1.52–1.88
>12 years	1.00	0.68–1.48	0.71	0.38–1.32	1.51	1.34–1.70	1.89	1.64–2.19
Disease duration	1.06	0.98–1.15	0.91	0.79–1.06	—	—	—	—
Living status								
Alone	1.00	—	1.00	—	1.00	—	1.00	—
With spouse or other family members	1.46	0.93–2.29	1.43	0.67–3.04	1.00	0.88–1.14	0.91	0.77–1.07
With others	0.87	0.57–1.33	0.56	0.27–1.17	0.58	0.51–0.66	0.70	0.58–8.33
Family support								
Adequate material and emotional support	1.00	—	1.00	—	1.00	—	1.00	—
Only material or emotional support	1.13	0.87–1.48	0.95	0.61–1.49	0.44	0.40–0.48	0.23	0.20–0.25
No material nor emotional support	0.56	0.26–1.22	1.27	0.48–3.40	0.29	0.23–0.37	0.22	0.14–0.34
No family or other condition	1.04	0.47–2.28	0.50	0.09–2.69	0.34	0.25–0.47	0.29	0.19–0.43

* Group I = a CHD with other chronic diseases group, Group II = a CHD only group, Group III = non-CHD patients with one (or more) chronic disease group, Group IV = non-patient group. CHD, coronary heart disease. Highlights in this table indicated that the effect size has reached a statistically significant level (*p* < 0.05).

## Discussion

This study was grounded in a large, long-term follow-up cohort study conducted among the elderly in China. Findings revealed a consistent decline in self-reported subjective sleep quality and health status as individuals aged. Notably, people with mild CHD (group I and II), regardless of comorbid conditions such as chronic pneumonia, diabetes, or hypertension, reported significantly lower self-assessed scores for both sleep quality and overall health status when compared to participants without CHD (group III and IV). Moreover, the older participants in a CHD only group (group II) exhibited a more rapid deterioration in their self-reported subjective sleep quality and health status when compared to those without CHD. Factors such as age, gender, education, disease duration, and family support were found to be potentially influence the self-reported subjective sleep quality in patients with mild CHD (group I or II). Conversely, older patients without CHD (group III and IV) had factors such as age, education level, living status, and family support potentially influence their self-assessed health status.

Firstly, individuals diagnosed with mild CHD in this study reported poorer sleep quality, overall health status, and had a faster rate of deterioration for both sleep quality and health status. This suggests a potential heightened psychological stress experienced by patients with mild CHD. Individuals with chronic diseases and who reside in care institutions, could be at a risk of developing a pessimistic attitude towards their own health status, which is similar to the findings of Osundolire et al. (2023). Vandiver et al. (2018) also found that the “community-based home caregiving model” has a positive impact on the quality of life for adults with chronic diseases. Multiple studies have also identified a higher prevalence of depression, anxiety, stress, and insomnia among patients with CHD when compared to the general adult population (Davidson et al., 2018; Eng et al., 2011). Aghaei et al. (2015) reported that stress and depression levels in patients with CHD exceeded those observed in cancer patients. Furthermore, a biometric analysis study has suggested that addressing negative emotions in patients with both CHD and other multiple comorbidities may represent a new frontier in future healthcare. This present study revealed that patients with both CHD and other chronic diseases showed poorer self-reported subjective sleep quality and health status, highlighting the need for increased attention to this group of people.

Secondly, this study observed that as the disease progressed, all patients with CHD (groups I and II) CHD exhibited improved self-reported subjective sleep quality. This may be attributed to the increased acceptance of the illness by patients with mild CHD. Many studies call for support in self-management of chronic disease patients, such as health education (Bodenheimer et al., 2002; Lorig and Holman, 2003). Building upon this finding, when faced with limitations in healthcare resources, it may be wise to prioritize interventions for newly diagnosed CHD cases, facilitating the efficient allocation of relevant healthcare resources. This observation aligns with many relevant studies. For example, a study on patients with chronic heart failure (CHF) showed that the acceptance of CHF significantly impacted a patient’s quality of life, with the levels of acceptance of CHF exhibiting a positive relationship with sleep quality (Obiegło et al., 2016). Moreover, other research has revealed that the acceptance of an illness is independently associated with almost every domain of a patients quality of life, such as emotional factors (depression and anxiety) (Marthoenis et al., 2021).

Thirdly, both non-CHD patients with one (or more) chronic disease group (group III) and non-patients (group IV) showed that education level had a significant positive correlation with both self-assessed sleep quality and health status. A possible reason for this correlation could be due to elderly with a better education level being more likely to ask for more social support from family and friends. A cross-sectional study revealed a connection between the education level, knowledge and attitudes were related to self-care in the management of chronic diseases (Borba et al., 2019). Therefore, it becomes imperative to consider interdisciplinary educational initiatives that encompass socioeconomic, psycho-emotional, and educational dimensions in order to ameliorate the negative emotions experienced by patients with CHD, while preserving their autonomy and functionality (Li et al., 2022). Several studies have already explored and yielded positive outcomes in this regard. Psychological interventions have been found to improve psychological symptoms, including depressive symptoms, anxiety, and stress, while also reducing cardiac mortality among individuals with CHD (Richards et al., 2018). Furthermore, a randomized controlled trial revealed that a transtheoretical model-based intervention and motivational interviewing could bring about positive changes in the stages of behavior, as well as higher scores for perceived benefits and self-efficacy, and lower perceived barriers and depression in participants (Li et al., 2020). A biometric analysis study spanning from 2004 to 2022 underscored the need for a multi-component and interdisciplinary approach. This would involve the collaboration of clinical psychologists, psychiatrists, dietitians, and exercise therapists to develop tailored psychosocial, pharmacological, and behavioral interventions for patients grappling with psycho-cardiological issues (Ge et al., 2022).

The present study was based on a Psycho-Cardiology model, it found that patients with mild CHD reported experiencing poorer sleep quality and health status. However, these conditions showed improvement as the disease progressed, alongside an increased acceptance of illness. Additionally, patients with mild CHD with higher levels of education demonstrated better sleep quality and health status. In light of these findings, it is imperative for healthcare providers, caregivers, and families to be vigilant about the psychological burden faced by patients with CHD and consider offering psycho-emotional interventions and appropriate support. Moreover, it is worth noting that the psychological impact at the time of the initial disease diagnosis may be the most profound, underscoring the importance of allocating additional care resources to newly diagnosed mild CHD cases, representing an innovative discovery.

Nonetheless, there are some limitations to this study. Firstly, our study is based on an open cohort design, and the follow-up periods for participants vary. Factors such as death and patients leaving the community for institutional care, can cause the loss of follow-up patients every year, with this kind of loss being a natural occurrence of the community population. To mitigate the potential bias stemming from missing data, strict statistical analysis methods were adopted. Secondly, this study was a single-center design, and the severity of CHD chosen was mild, so our results can provide public health evidence for the intervention strategy of mild CHD community patients. However, any interventions formulated would need to consider cultural and geographical circumstances. Prior research has suggested a potential negative relationship between the ability to regulate emotions and the severity of coronary stenosis (Shi et al., 2022). Thirdly, the present study adopted an observational cohort design, with the participants being in the natural state. Therefore, it did not provide any intervention to participants. Lastly, this study collected self-reported data, which may have increased the risk of biased results when compared to structured interviews conducted by experts. However, this bias could be reduced by the large sample size. Meantime, this study used some non-normed single items, which may threaten the validity of the findings. Therefore, future efforts would focus on incorporating the severity of disease, objective health measures, and a more detailed exploration of intervention strategies.

## Conclusion

The present study based on the data of 44,552 elderly people, and 9-year follow-up cohort design. Identified that patients with mild CHD in a community exhibited lower self-reported subjective sleep quality, health status scores and a more rapid health-sleep decline, suggesting a heightened level of psychological stress within this population. Moreover, higher levels of education appeared to serve as a protective factor against psychological stress. This underscores the importance of implementing psycho-emotional interventions, awareness campaigns, and educational measures for patients with mild CHD. Moreover, patients with CHD had better sleep quality as they aged and the disease progressed. This is of particular significance, as the consideration of newly diagnosed CHD cases should be a priority group for early intervention, with the aim of helping patients having a higher chance of accepting their illness early on. This study offers evidence for the management of patients with mild CHD and informs the efficient utilization of relevant healthcare resources.

## Data Availability

The original contributions presented in the study are included in the article/supplementary material, further inquiries can be directed to the corresponding authors.

## Appendix

Table A1.

**Table A1:** Descriptive data of variables.

Characteristics	Mean	SD	Skewness	Kurtosis	*p**
Age	74.90	10.35	−1.12	0.22	<0.001
Disease duration	2.15	1.29	0.23	1.02	<0.001
Self-reported subjective sleep quality score	2.90	1.07	−0.68	−0.23	<0.001
Self-assessed health score	3.36	0.91	−0.02	−0.04	<0.001

SD, standard deviation. ^*****^ *p* value of normality using Kolmogorov-Smirnov test.
